# State-of-the-art and novel approaches to mild solubilization of inclusion bodies

**DOI:** 10.3389/fbioe.2023.1249196

**Published:** 2023-07-20

**Authors:** Robert Klausser, Julian Kopp, Eva Prada Brichtova, Florian Gisperg, Mohamed Elshazly, Oliver Spadiut

**Affiliations:** ^1^ Research Division Integrated Bioprocess Development, Institute of Chemical, Environmental and Bioscience, Vienna, Austria; ^2^ Christian Doppler Laboratory IB Processing 4.0, Technische Universität Wien, Vienna, Austria

**Keywords:** inclusion bodies, mild solubilization, ionic liquids, molecular dynamics simulation, refolding, aggregates

## Abstract

Throughout the twenty-first century, the view on inclusion bodies (IBs) has shifted from undesired by-products towards a targeted production strategy for recombinant proteins. Inclusion bodies can easily be separated from the crude extract after cell lysis and contain the product in high purity. However, additional solubilization and refolding steps are required in the processing of IBs to recover the native protein. These unit operations remain a highly empirical field of research in which processes are developed on a case-by-case basis using elaborate screening strategies. It has been shown that a reduction in denaturant concentration during protein solubilization can increase the subsequent refolding yield due to the preservation of correctly folded protein structures. Therefore, many novel solubilization techniques have been developed in the pursuit of mild solubilization conditions that avoid total protein denaturation. In this respect, ionic liquids have been investigated as promising agents, being able to solubilize amyloid-like aggregates and stabilize correctly folded protein structures at the same time. This review briefly summarizes the state-of-the-art of mild solubilization of IBs and highlights some challenges that prevent these novel techniques from being yet adopted in industry. We suggest mechanistic models based on the thermodynamics of protein unfolding with the aid of molecular dynamics simulations as a possible approach to solve these challenges in the future.

## 1 Mild inclusion body solubilization

While initially inclusion bodies (IBs) were thought to be aggregates of misfolded protein (García-Fruitós et al., 2012), they have been shown to contain correctly folded secondary and tertiary protein structures, in some cases even with biological activity (García-Fruitós, 2010; Villaverde et al., 2015; Belkova et al., 2022; López-Cano et al., 2022). Preserving these existing structures during the solubilization step has been correlated with an increase in refolding yield due to reduced re-aggregation (Singh et al., 2015; Kachhawaha et al., 2022). Thus, the concept of “mild solubilization” originated, describing the solubilization of IBs without fully denaturing the protein. Many researchers use an arbitrary maximum threshold of 2–3 M Urea to demarcate “mild” from “traditional” solubilization (Singh et al., 2015; Upadhyay et al., 2016; Nekoufar et al., 2020; Maksum et al., 2022; Mohammadi et al., 2023). However, the degree of denaturation induced by these conditions is highly dependent on the respective protein of interest (PoI). Therefore, in this manuscript, “mild solubilization” describes attempts to solubilize aggregated protein in such a way that all existing misfolded structures are unfolded, whilst the highest possible amount of already existing correctly folded secondary structures are preserved.

Despite the advantages of mild solubilization, it is still necessary to add a sufficient amount of denaturing agent during solubilization to allow misfolded structures to unfold. Hall et al. (2018) showed that dimeric disulfide-linked recombinant human bone morphogenic protein-2 could be extracted from IBs without denaturation by using a buffer containing 4 M urea and 250 mM guanidinium hydrochloride (Gnd-HCl). However, the extracted protein showed no bioactivity. It was hypothesized that this was due to the incorrectly folded disulfide bridges and hydrophobic core. This was supported by refolding the protein after solubilization at 6 M Gnd-HCl, thereby producing a bioactive product. This example shows that successful mild solubilization has to balance preserving the correctly folded structures with the unfolding of any misfolded structures.

Several analytical methods have been established to evaluate the structural changes during the solubilization and subsequent refolding of IBs. Infrared (IR) and Raman spectroscopy can be used to track changes in the secondary and tertiary structure of proteins, as well as the formation of disulfide bonds (Pauk et al., 2021). FT-IR spectroscopy can even be used to differentiate and quantify intramolecular α-helix structures from amyloid-like β-sheet bridges of IBs within intact cells (Ami et al., 2006). By deconvoluting the IR spectra, the contributions of individual structure types, such as α-helices or β-sheets, can be quantified (Umetsu et al., 2005). However, amide signals of the protein commonly overlap with prominent water and urea signals. Therefore, high protein concentrations are required to use IR and Raman spectroscopy.

Another spectroscopic method for protein structure analysis is circular dichroism (CD) spectroscopy (Clarke, 2011), which measures the difference in absorption of right- and left-circularly polarized light. The resulting spectra show characteristic bands for the individual secondary protein structure types.

If the (un)folding occurs as a two-state reaction, differential scanning calorimetry (DSC) can be used to measure the enthalpy of unfolding (Ionescu et al., 2008). This is especially important to characterize the thermodynamics of the folding states.

Dynamic light scattering (DLS) is a widely used method to measure particle size distribution. This information can be used to track aggregation processes. Moreover, this technique can also be used to measure the hydrodynamic radius of proteins. As the protein unfolds, its hydrodynamic radius increases, thus enabling DLS to monitor the unfolding process (Yu et al., 2013).

Finally, intrinsic tryptophan (= Trp) fluorescence is a very robust method often used to track *in situ* refolding. The observed fluorescence maximum shifts as Trp residues in the protein get buried within the hydrophobic core during the folding process (Duy and Fitter, 2006). Additionally, acrylamide quenches Trp fluorescence via an entirely physical mechanism. This can be used to determine the Stern-Volmer constant and therefore quantify the amount of Trp residues located within the core of the protein *versus* those positioned towards the bulk medium (Tallmadge et al., 1989; Upadhyay et al., 2016).

In many protein folding studies, CD spectroscopy is used as a secondary analysis method alongside intrinsic Trp fluorescence. As a larger number of structural groups (amide bonds, aromatic amino acids, disulfide bonds) contribute to the information gained (Clarke, 2011; Pauk et al., 2021), CD spectroscopy is able to give detailed information about the structure of the protein. Meanwhile, Trp fluorescence offers high sensitivity and is compatible with high solute concentrations. These traits also make Trp fluorescence spectroscopy an excellent option to be considered as an online PAT tool for solubilization and refolding processes.

To achieve mild solubilization without total denaturation of the protein, reduced amounts of the traditional solubilizing agents, urea and Gnd-HCl, are usually paired with other chemical or physical conditions. Most prominently, alkaline pH is used to increase protein solubility at lower denaturant concentrations (Khan et al., 1998; Patra et al., 2000; Singh et al., 2008; Lu and Lin, 2012; Ishikawa et al., 2022). Other chemical options are organic solvents (Upadhyay et al., 2016; Sarker et al., 2019; Nekoufar et al., 2020) or detergents, like N-lauroyl sarcosine, SDS, CHAPS, and Triton X-100 (Francis et al., 2012; Ishikawa et al., 2022; López-Cano et al., 2022). Physical methods for mild solubilization include high pressures of up to 2.4 kbar (Chura-Chambi et al., 2022a; Chura-Chambi et al., 2022b) and, most recently, freeze-thaw cycles (Padhiar et al., 2018; Maksum et al., 2022). Finally, ionic liquids (ILs) are novel solvents able to dissolve IBs whilst potentially retaining secondary or even tertiary protein structure.

### 1.1 Ionic liquids as mild solubilization agents

ILs are salts in a liquid state at temperatures below 100°C that have gained much attention in IB processing due to their adaptable physicochemical properties and environmental benefits. ILs have shown great potential as mild solubilization agents, dissolving aggregated protein whilst preserving native secondary structures of proteins (Fujita et al., 2016). Furthermore, they can help refold chemically denatured protein by replacing urea or Gnd-HCl from its solvation layer due to preferential interaction (Singh and Patel, 2018; Sindhu et al., 2020). Both of these properties could help to intensify refolding processes and lower their environmental burden by reducing the need of diluting the solubilizate. The influence of the most common IL ions on protein folding has been recently reviewed by Guncheva (2022).

Similarly, deep eutectic solvents (DES) are a subclass of ILs which has been recently investigated in protein stability studies (Yadav and Venkatesu, 2022). DES are mixtures of salts that are liquid at room temperature since the eutectic mixture has a significantly lower melting point compared to the individual components (Abbott et al., 2003). Although there have been several interesting studies concerning the conformational stability and folding state of proteins in these solvents (Niknaddaf et al., 2018; Kist et al., 2019; Sanchez-Fernandez et al., 2022), to our knowledge, there is no literature available yet regarding the solubilization of aggregated protein in DES. This emerging field of research is important for industrial applications, as many DES are comprised of cheap and biocompatible bulk chemicals (Gonçalves et al., 2021; Jesus et al., 2023; Usmani et al., 2023). Furthermore, DES are capable of being recycled, providing an economical and environmental advantage over traditional chaotropic agents (Prabhune and Dey, 2023).

**FIGURE 1:**
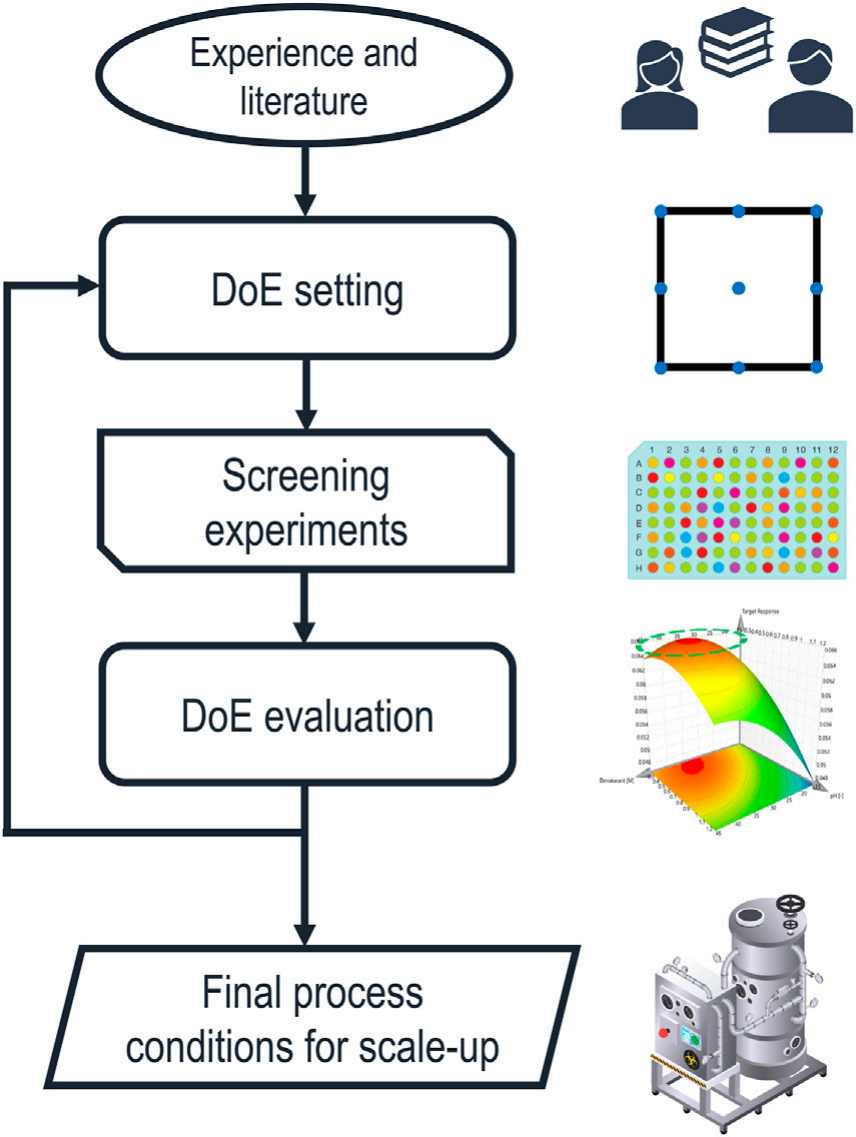
Schematic representation of the established approach to solubilization process development. The factors of the first statistical design of experiments (DoE) are chosen based on experience and literature, while the ranges are limited by the technical feasibility of the screening experiments. After evaluating the results, the DoE is iterated with adapted design spaces until sufficiently optimized process parameters are found and transferred to larger scales.

Despite these potential benefits, ILs and DES are very challenging to fit into the currently established IB process design strategy. The early development of the chemical environment for solubilization and refolding is still carried out by empirical and elaborate screening experiments, as summarized in a recent review (Buscajoni et al., 2022). A schematic depiction of the currently established process development steps is shown in Figure 1.

The initial selection of buffer components is based on experience and reviewing the literature. Therefore, the design space is usually constrained to a short list of already established chemicals. Screening experiments are generally done in a DoE approach, maximizing responses, such as the solubilization efficiency, final product concentration after refolding, or the refolding yield, using the response surface method (Ahmadian et al., 2020). This approach can be iterated with adapted design spaces until an optimum for the desired response is found. The resulting model is then used to define the process parameters. Alternatively, data-driven models can predict optimal process parameters based on experimental data. A recent publication (Walther et al., 2022) has shown such a data-driven integrated process model for the solubilization, refolding, and purification steps in an industrial setting. This empiric approach based on statistics efficiently optimizes a set of quantitative process parameters. Still, the choice of the initial design space, based on experience, is highly influential on the final process, and the results cannot be transferred between different PoIs. Furthermore, if a comprehensive list of chemicals and their combinations are considered as buffer components, the experimental designs become very expensive in time and resources.

Besides some technical constraints (i.e., high pressure), this seems to be one of the reasons that, besides alkaline pH, none of the developed mild solubilization techniques are applied in industrial processes yet. Especially ILs are very hard to integrate into the screening-based approach due to the sheer amount of possible ion pair combinations. Another disadvantage of the current design method is the lack of generated platform knowledge. Therefore, this cumbersome and time-intensive process has to be repeated for each new PoI. The missing platform knowledge is also problematic when the push toward quality by design (QbD) principles is considered (ICH, 2017; Beg et al., 2019).

One approach to generate this knowledge is the formulation of mathematical models for each process step. However, for the solubilization and refolding steps, there is still a lack of mechanistic models describing the effects of the chemical environment. The refolding step is usually described by kinetic models, parametrizing reaction rates from the denatured to intermediate, native, or aggregated states (Jungbauer and Kaar, 2007; Pauk et al., 2021). The kinetics of solubilization has been shown to be predominantly dependent on pore diffusion resistance into the IB particles (Walther et al., 2013; Walther et al., 2014). However, while these kinetic models present an important tool to describe the influence of factors like protein concentration and process times, they do not help in choosing a suitable buffer composition for mild solubilization. To address these shortcomings of the currently established process design approach, the authors want to suggest the use of two theoretical tools:

1) Thermodynamic unfolding models as a way to describe and predict the solubilization process more precisely, and
2) Molecular dynamics simulations (MDS) to predict the interactions of the PoI with a wide array of chemicals.

## 2 Mechanistic models and molecular dynamics simulations for solubilization prediction

While no mechanistic models specifically describing the solubilization of IBs have been published so far, the thermodynamics of protein folding has been studied extensively. The chemical denaturation of a protein in an aqueous system occurs because a denaturant preferentially binds to the protein over water. Since the unfolded state provides a greater number of interaction sites for the denaturant, this confirmation is energetically favored, and the protein unfolds. Early observations showed that the free energy of unfolding in water linearly correlates to the denaturant concentration (Pace et al., 2008). This linearity can be explained by the protein-solvent interaction behaving more similarly to a solvent exchange than to covalent binding (Schellman, 2002; Pace et al., 2008). The slope of this system-specific linear correlation is called the “m-value” (Greene and Pace, 1974) and can be used to estimate the free enthalpy of unfolding within water. While this m-value helps quantify “denaturation power” in a system (Magsumov et al., 2020), it is still an empiric parameter without a clear mechanistic interpretation (Wakayama et al., 2019). To formulate a mechanistic model, physically defined parameters are required, as exemplarily proposed by Hall et al. (2018). This model describes chemical denaturation via six parameters, representing a countable number of interaction sites for both the folded and unfolded state, distinctive binding constants for different groups of interaction sites, and the intrinsic stability of the native protein structure. However, this model was still built on insights gained from denaturation experiments using urea and Gnd-HCl as denaturants. Among other aspects, Wakayama et al. (2019) expanded the existing thermodynamic models by considering the possibility of stronger binding interactions and alternative denaturing factors, such as high pressure or temperature, thus, potentially creating a mechanistic basis for physical methods of mild solubilization and new denaturants like ILs.

However, this model still does not resolve the problem of choosing a suitable denaturant without screening all options, as the model parameters are always specific for a definite protein-solvent system. Instead of limiting the possibilities to urea and Gnd-HCl, it would be required to estimate a protein’s solubility in a wide range of alternative solvents, being especially relevant to ILs.

The solubility of protein can be partly estimated using the Hofmeister series (Hofmeister, 1888), which has developed into a series comparing individual cations and anions, qualitatively describing their influence on protein solubility. These Hofmeister effects were initially attributed to the ions increasing or decreasing the H-bond structuring of water. The H-bond structure was assumed to affect the hydration layer of the protein, explaining the influence on solubility. While this is partly true, extensive research revealed that the actual mechanisms are a far more complex mixture of Coulombic and disperse interactions, excellently reviewed by Schröder (2017) within the context of ILs. These newer mechanistic insights into protein-solvent interactions are heavily based on computational science, especially MDS. These simulations have been used to quantify the effect of different ions on the solubility of hydrophobic solutes (Thomas and Elcock, 2007), thereby differentiating the term “Hofmeister effects” into three categories of individually quantifiable contributions:

1) water—water hydrogen bonding (or “water structuring” in the Hofmeister context).
2) free energy of the hydrophobic interaction between solutes and
3) preferential interaction of ions and solutes.

**FIGURE 2:**
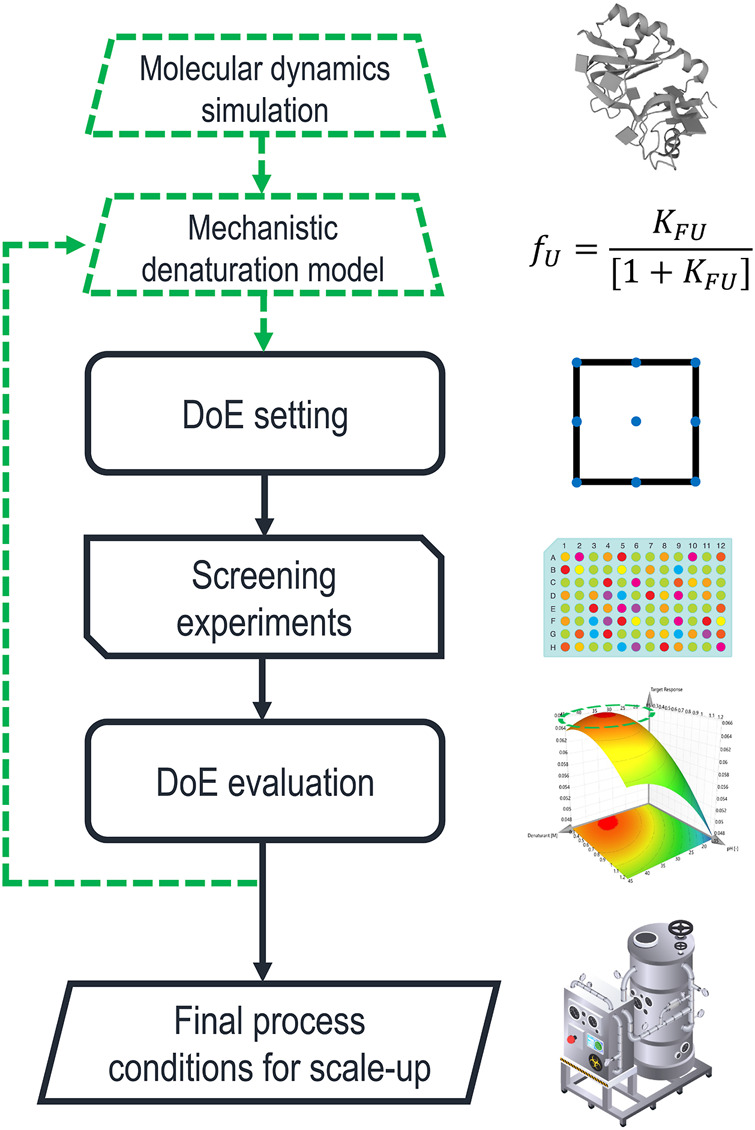
Schematic description of solubilization process development including the proposed additions highlighted by green dashed lines. Molecular dynamics simulations (MDS) are used to determine a first estimation for the parameters of a mechanistic model describing protein denaturation. Based on these values, a range of promising denaturants and buffer components are picked for the initial experimental design. The iterative optimization loop feeds back into the denaturation model, which is used as an input for the next design space.

To investigate the specific interaction of individual solvent molecules with proteins, MDS have become state-of-the-art (Schröder, 2017; Ferina and Daggett, 2019; Otzen et al., 2022; Sinha et al., 2022). In these simulations, the motions of a protein molecule and the surrounding solvent molecules are simulated under the influence of their respective force fields. MDS have been used to gain insight into the distance and orientation between proteins and other solutes/solvents (de Oliveira and Martínez, 2020; Otzen et al., 2022), protein-protein interactions, such as aggregation (Loureiro and Faísca, 2020), as well as preferred interaction tendencies between multi-component mixtures (Ghosh et al., 2017). These insights might be used to formulate an early prediction of suitable chemical environments to solubilize IBs, without the need to conduct a single experiment in the lab. Figure 2 shows a potential extension of the currently established process development approach using the suggested methods.

MDS could be used to investigate the protein-solvent interactions for a comprehensive list of chemicals. These simulations could give a first estimation of the parameters that describe the denaturation curve, determining the concentration range in which the PoI is partially folded. Thus, novel solubilizing agents could be considered without additional screening experiments in the laboratory. Furthermore, basing experimental designs on physical parameters could be a key step to generate transferable process knowledge, leading to platform technology and QbD. While the significant computational cost of MDS must be considered (Sinha et al., 2022), there have been significant recent advances limiting this downside. Besides methodological improvements (Dominic et al., 2023), cloud-computing approaches (Zimmerman et al., 2021) and artificial neural networks (Tsai et al., 2022; Dominic et al., 2023) are significantly improving real-time efficiency. Furthermore, the optimization of simulation software for graphics processing units (GPUs) has made MDS feasible for consumer-grade hardware (Hollingsworth and Dror, 2018). The impact of GPU technology on the availability of MDS can be illustrated by comparing two studies that benchmark the hardware available at the time of their publication. In 2013 an NVIDIA GTX-TITAN was able to simulate dihydrofolate reductase, a 21.5 kDa protein comprised of 23,558 atoms, for 110.65 ns per day of computation (Salomon-Ferrer et al., 2013). Six years later, an NVIDIA RTX 2080 generated the same simulation times for an 80,000-atom membrane protein embedded in a lipid bilayer, including the surrounding water and ion molecules (Kutzner et al., 2019). In comparison, a recent study by Piccoli and Martínez (2022) used MDS to predict the denaturing effects of four ILs on ubiquitin by generating 3D structures of denatured ubiquitin with two longer simulations of 50 and 100 ns, respectively. Then MDS of only 10 ns each could be used to investigate the interactions of the ILs with the protein in different folding states. Considering these simulation times, current consumer hardware is likely to be powerful enough for the proposed use of MDS.

## 3 Conclusion and outlook

The mild solubilization approach aims to preserve protein structures present in IBs to increase refolding yields by reducing the reaggregation of the solubilized PoI. This has been empirically done by reducing urea and Gnd-HCl concentrations during the solubilization while using alkaline pH, high pressure, detergents, organic solvents, and freeze-thaw cycles to increase protein solubility. Recently, ILs have been investigated, both as a very promising method to solubilize protein aggregates without unfolding their native-like structure, as well as refolding additives that counteract the effects of the traditional denaturants. However, due to the number of possible ion combinations, the established process design method of empirically screening buffer components quickly leads to an overwhelming number of experiments. Furthermore, this approach does not generate platform knowledge and has to be repeated for each new PoI.

To improve the established design process, MDS could be used in combination with mechanistic models that describe the thermodynamics of protein (un)folding to base experimental designs on physical parameters and improve process understanding. The current advances in the field of machine learning, and algorithms like AlphaFold, have made significant progress toward sequence-based protein structure prediction (Nussinov et al., 2022). These novel tools could soon be used to obtain the main requirement for MDS, a detailed 3D structure of the protein, without resource-intensive protein structure analytics. Combining these knowledge-based simulation tools might even enable the prediction of an entire refolding process based on the sequence of the PoI.

## References

[B1] Abbott A. P. Capper G. Davies D. L. Rasheed R. K. Tambyrajah V. (2003). Novel solvent properties of choline chloride/urea mixtures. Chem. Commun. 7, 70–71. 10.1039/b210714g 12610970

[B2] Ahmadian M. Jahanian-Najafabadi A. Akbari V. (2020). Optimization of buffer additives for efficient recovery of hGM-CSF from inclusion bodies using response surface methodology. IJPR 19, 297–309. 10.22037/ijpr.2020.1101169 33680031 PMC7758011

[B3] Ami D. Natalello A. Taylor G. Tonon G. Maria Doglia S. (2006). Structural analysis of protein inclusion bodies by Fourier transform infrared microspectroscopy. Biochimica Biophysica Acta (BBA) - Proteins Proteomics 1764, 793–799. 10.1016/j.bbapap.2005.12.005 16434245

[B4] Beg S. Hasnain M. S. Rahman M. Swain S. (2019). “Introduction to quality by design (QbD): Fundamentals, principles, and applications,” in Pharmaceutical quality by design (Netherlands: Elsevier), 1–17. 10.1016/B978-0-12-815799-2.00001-0

[B5] Belkova M. Köszagová R. Nahálka J. (2022). Active inclusion bodies: The unexpected journey. J. Microb. Biotech. food Sci. 2022, e5951. 10.55251/jmbfs.5951

[B6] Buscajoni L. Martinetz M. C. Berkemeyer M. Brocard C. (2022). Refolding in the modern biopharmaceutical industry. Biotechnol. Adv. 61, 108050. 10.1016/j.biotechadv.2022.108050 36252795

[B7] Chura-Chambi R. M. Farah C. S. Morganti L. (2022a). Human growth hormone inclusion bodies present native-like secondary and tertiary structures which can be preserved by mild solubilization for refolding. Microb. Cell Fact. 21, 164. 10.1186/s12934-022-01887-1 35978337 PMC9382763

[B8] Chura-Chambi R. M. Prieto-da-Silva A. R. B. Di Lela M. M. Oliveira J. E. Abreu P. E. A. Meireles L. R. (2022b). High level SARS-CoV-2 nucleocapsid refolding using mild condition for inclusion bodies solubilization: Application of high pressure at pH 9.0. PLoS ONE 17, e0262591. 10.1371/journal.pone.0262591 35113919 PMC8812862

[B9] Clarke D. T. (2011). “Circular dichroism and its use in protein-folding studies,” in Protein folding, misfolding, and disease methods in molecular biology. Editors Hill A. F. Barnham K. J. Bottomley S. P. Cappai R. (Totowa, NJ: Humana Press), 59–72. 10.1007/978-1-60327-223-0_5 21713631

[B10] de Oliveira I. P. Martínez L. (2020). The shift in urea orientation at protein surfaces at low pH is compatible with a direct mechanism of protein denaturation. Phys. Chem. Chem. Phys. 22, 354–367. 10.1039/C9CP05196A 31815262

[B11] Dominic A. J. Cao S. Montoya-Castillo A. Huang X. (2023). Memory unlocks the future of biomolecular dynamics: Transformative tools to uncover physical insights accurately and efficiently. J. Am. Chem. Soc. 145, 9916–9927. 10.1021/jacs.3c01095 37104720

[B12] Duy C. Fitter J. (2006). How aggregation and conformational scrambling of unfolded states govern fluorescence emission spectra. Biophysical J. 90, 3704–3711. 10.1529/biophysj.105.078980 PMC1440751 16500981

[B13] Ferina J. Daggett V. (2019). Visualizing protein folding and unfolding. J. Mol. Biol. 431, 1540–1564. 10.1016/j.jmb.2019.02.026 30840846

[B14] Francis V. G. Majeed M. A. Gummadi S. N. (2012). Recovery of functionally active recombinant human phospholipid scramblase 1 from inclusion bodies using N -lauroyl sarcosine. J. Industrial Microbiol. Biotechnol. 39, 1041–1048. 10.1007/s10295-012-1105-1 22389205

[B15] Fujita K. Kajiyama M. Liu Y. Nakamura N. Ohno H. (2016). Hydrated ionic liquids as a liquid chaperon for refolding of aggregated recombinant protein expressed in *Escherichia coli* . Chem. Commun. 52, 13491–13494. 10.1039/C6CC06999A 27801474

[B16] García-Fruitós E. (2010). Inclusion bodies: A new concept. Microb. Cell Fact. 9, 80. 10.1186/1475-2859-9-80 21040537 PMC2987918

[B17] García-Fruitós E. Vázquez E. Díez-Gil C. Corchero J. L. Seras-Franzoso J. Ratera I. (2012). Bacterial inclusion bodies: Making gold from waste. Trends Biotechnol. 30, 65–70. 10.1016/j.tibtech.2011.09.003 22037492

[B18] Ghosh S. Dey S. Patel M. Chakrabarti R. (2017). Can an ammonium-based room temperature ionic liquid counteract the urea-induced denaturation of a small peptide? Phys. Chem. Chem. Phys. 19, 7772–7787. 10.1039/C6CP08842B 28262899

[B19] Gonçalves A. R. P. Paredes X. Cristino A. F. Santos F. J. V. Queirós C. S. G. P. (2021). Ionic liquids—a review of their toxicity to living organisms. IJMS 22, 5612. 10.3390/ijms22115612 34070636 PMC8198260

[B20] Greene R. F. Pace C. N. (1974). Urea and guanidine hydrochloride denaturation of ribonuclease, lysozyme, α-chymotrypsin, and b-lactoglobulin. J. Biol. Chem. 249, 5388–5393. 10.1016/S0021-9258(20)79739-5 4416801

[B21] Guncheva M. (2022). Role of ionic liquids on stabilization of therapeutic proteins and model proteins. Protein J. 41, 369–380. 10.1007/s10930-022-10058-5 35661292

[B22] Hall D. Kinjo A. R. Goto Y. (2018). A new look at an old view of denaturant induced protein unfolding. Anal. Biochem. 542, 40–57. 10.1016/j.ab.2017.11.011 29158130

[B23] Hofmeister F. (1888). Zur Lehre von der Wirkung der Salze: Zweite Mittheilung. Arch. F. Exp. Pathol. U. Pharmakol 24, 247–260. 10.1007/BF01918191

[B24] Hollingsworth S. A. Dror R. O. (2018). Molecular dynamics simulation for all. Neuron 99, 1129–1143. 10.1016/j.neuron.2018.08.011 30236283 PMC6209097

[B25] ICH (2017). “International conference on harmonisation of technical requirements for registration of pharmaceuticals for human use,” in ICH guideline Q8 (R2) on pharmaceutical development (Netherlands: European Medicine Agency).

[B26] Ionescu R. M. Vlasak J. Price C. Kirchmeier M. (2008). Contribution of variable domains to the stability of humanized IgG1 monoclonal antibodies. J. Pharm. Sci. 97, 1414–1426. 10.1002/jps.21104 17721938

[B27] Ishikawa S. Ishikawa H. Sato A. (2022). Improved refolding of a human IgG1 Fc (CH2-CH3) scaffold from its inclusion body in <i&gt;*E. coli*&lt;/i&gt; by alkaline solubilization. Biol. Pharm. Bull. 45, 284–291. 10.1248/bpb.b21-00796 35228394

[B28] Jesus A. R. Paiva A. Duarte A. R. C. (2023). Current developments and future perspectives on biotechnology applications of natural deep eutectic systems. Curr. Opin. Green Sustain. Chem. 39, 100731. 10.1016/j.cogsc.2022.100731

[B29] Jungbauer A. Kaar W. (2007). Current status of technical protein refolding. J. Biotechnol. 128, 587–596. 10.1016/j.jbiotec.2006.12.004 17222934

[B30] Kachhawaha K. Singh S. Joshi K. Nain P. Singh S. K. (2022). Bioprocessing of recombinant proteins from *Escherichia coli* inclusion bodies: Insights from structure-function relationship for novel applications. Prep. Biochem. Biotechnol. 2022, 1–25. 10.1080/10826068.2022.2155835 36534636

[B31] Khan R. H. Appa Rao K. B. C. Eshwari A. N. S. Totey S. M. Panda A. K. (1998). Solubilization of recombinant ovine growth hormone with retention of native-like secondary structure and its refolding from the inclusion bodies of *Escherichia coli* . Biotechnol. Prog. 14, 722–728. 10.1021/bp980071q 9758661

[B32] Kist J. A. Henzl M. T. Bañuelos J. L. Baker G. A. (2019). Calorimetric evaluation of the operational thermal stability of ribonuclease A in hydrated deep eutectic solvents. ACS Sustain. Chem. Eng. 7, 12682–12687. 10.1021/acssuschemeng.9b02585

[B33] Kutzner C. Páll S. Fechner M. Esztermann A. Groot B. L. Grubmüller H. (2019). More bang for your buck: Improved use of GPU nodes for GROMACS 2018. J. Comput. Chem. 40, 2418–2431. 10.1002/jcc.26011 31260119

[B34] López-Cano A. Sicilia P. Gaja C. Arís A. Garcia-Fruitós E. (2022). Quality comparison of recombinant soluble proteins and proteins solubilized from bacterial inclusion bodies. New Biotechnol. 72, 58–63. 10.1016/j.nbt.2022.09.003 36150649

[B35] Loureiro R. J. S. Faísca P. F. N. (2020). The early phase of β2-microglobulin aggregation: Perspectives from molecular simulations. Front. Mol. Biosci. 7, 578433. 10.3389/fmolb.2020.578433 33134317 PMC7550760

[B36] Lu S.-C. Lin S.-C. (2012). Recovery of active N-acetyl-d-glucosamine 2-epimerase from inclusion bodies by solubilization with non-denaturing buffers. Enzyme Microb. Technol. 50, 65–70. 10.1016/j.enzmictec.2011.09.010 22133442

[B37] Magsumov T. Ziying L. Sedov I. (2020). Comparative study of the protein denaturing ability of different organic cosolvents. Int. J. Biol. Macromol. 160, 880–888. 10.1016/j.ijbiomac.2020.05.260 32497668

[B38] Maksum I. P. Yosua Y. Nabiel A. Pratiwi R. D. Sriwidodo S. Soedjanaatmadja U. M. S. (2022). Refolding of bioactive human epidermal growth factor from *E. coli* BL21(DE3) inclusion bodies & evaluations on its *in vitro* & *in vivo* bioactivity. Heliyon 8, e09306. 10.1016/j.heliyon.2022.e09306 35497033 PMC9039848

[B39] Mohammadi M. Ghanbari S. Emamgholi A. Hashemzadeh M. S. (2023). Utilization of freeze-thawing method for high-level expression of functional human epidermal growth factor (hEGF). Int. J. Pept. Res. Ther. 29, 38. 10.1007/s10989-023-10510-9

[B40] Nekoufar S. Fazeli A. Fazeli M. R. (2020). Solubilization of human interferon β-1b inclusion body proteins by organic solvents. Adv. Pharm. Bull. 10, 233–238. 10.34172/apb.2020.027 32373491 PMC7191233

[B41] Niknaddaf F. Shahangian S. S. Heydari A. Hosseinkhani S. Sajedi R. H. (2018). Deep eutectic solvents as a new generation of chemical chaperones. ChemistrySelect 3, 10603–10607. 10.1002/slct.201802235

[B42] Nussinov R. Zhang M. Liu Y. Jang H. (2022). AlphaFold, artificial intelligence (AI), and allostery. J. Phys. Chem. B 126, 6372–6383. 10.1021/acs.jpcb.2c04346 35976160 PMC9442638

[B43] Otzen D. E. Pedersen J. N. Rasmussen H. Ø. Pedersen J. S. (2022). How do surfactants unfold and refold proteins? Adv. Colloid Interface Sci. 308, 102754. 10.1016/j.cis.2022.102754 36027673

[B44] Pace C. N. Grimsley G. R. Scholtz J. M. (2008). “Denaturation of proteins by urea and guanidine hydrochloride,” in Protein science encyclopedia. Editor Fersht A. R. (Weinheim, Germany: Wiley-VCH Verlag GmbH & Co KGaA), sf03. 10.1002/9783527610754.sf03

[B45] Padhiar A. A. Chanda W. Joseph T. P. Guo X. Liu M. Sha L. (2018). Comparative study to develop a single method for retrieving wide class of recombinant proteins from classical inclusion bodies. Appl. Microbiol. Biotechnol. 102, 2363–2377. 10.1007/s00253-018-8754-6 29387954

[B46] Patra A. K. Mukhopadhyay R. Mukhija R. Krishnan A. Garg L. C. Panda A. K. (2000). Optimization of inclusion body solubilization and renaturation of recombinant human growth hormone from *Escherichia coli* . Protein Expr. Purif. 18, 182–192. 10.1006/prep.1999.1179 10686149

[B47] Pauk J. N. Raju Palanisamy J. Kager J. Koczka K. Berghammer G. Herwig C. (2021). Advances in monitoring and control of refolding kinetics combining PAT and modeling. Appl. Microbiol. Biotechnol. 105, 2243–2260. 10.1007/s00253-021-11151-y 33598720 PMC7954745

[B48] Piccoli V. Martínez L. (2022). Ionic liquid solvation of proteins in native and denatured states. J. Mol. Liq. 363, 119953. 10.1016/j.molliq.2022.119953

[B49] Prabhune A. Dey R. (2023). Green and sustainable solvents of the future: Deep eutectic solvents. J. Mol. Liq. 379, 121676. 10.1016/j.molliq.2023.121676

[B50] Salomon-Ferrer R. Götz A. W. Poole D. Le Grand S. Walker R. C. (2013). Routine microsecond molecular dynamics simulations with AMBER on GPUs 2, explicit solvent particle mesh ewald. J. Chem. Theory Comput. 9, 3878–3888. 10.1021/ct400314y 26592383

[B51] Sanchez-Fernandez A. Basic M. Xiang J. Prevost S. Jackson A. J. Dicko C. (2022). Hydration in deep eutectic solvents induces non-monotonic changes in the conformation and stability of proteins. J. Am. Chem. Soc. 144, 23657–23667. 10.1021/jacs.2c11190 36524921 PMC9801427

[B52] Sarker A. Rathore A. S. Gupta R. D. (2019). Evaluation of scFv protein recovery from *E. coli* by *in vitro* refolding and mild solubilization process. Microb. Cell Fact. 18, 5. 10.1186/s12934-019-1053-9 30642336 PMC6330739

[B53] Schellman J. A. (2002). Fifty years of solvent denaturation. Biophys. Chem. 96, 91–101. 10.1016/S0301-4622(02)00009-1 12034431

[B54] Schröder C. (2017). Proteins in ionic liquids: Current status of experiments and simulations. Top. Curr. Chem. (Z) 375, 25. 10.1007/s41061-017-0110-2 PMC5480425 28176271

[B55] Sindhu A. Bhakuni K. Sankaranarayanan K. Venkatesu P. (2020). Implications of imidazolium-based ionic liquids as refolding additives for urea-induced denatured serum albumins. ACS Sustain. Chem. Eng. 8, 604–612. 10.1021/acssuschemeng.9b06194

[B56] Singh A. Upadhyay V. Upadhyay A. K. Singh S. M. Panda A. K. (2015). Protein recovery from inclusion bodies of *Escherichia coli* using mild solubilization process. Microb. Cell Fact. 14, 41. 10.1186/s12934-015-0222-8 25889252 PMC4379949

[B57] Singh S. M. Upadhyay A. K. Panda A. K. (2008). Solubilization at high pH results in improved recovery of proteins from inclusion bodies of *E. coli* . J. Chem. Technol. Biotechnol. 83, 1126–1134. 10.1002/jctb.1945

[B58] Singh U. K. Patel R. (2018). Dynamics of ionic liquid-assisted refolding of denatured cytochrome c: A study of preferential interactions toward renaturation. Mol. Pharm. 15, 2684–2697. 10.1021/acs.molpharmaceut.8b00212 29767978

[B59] Sinha S. Tam B. Wang S. M. (2022). Applications of molecular dynamics simulation in protein study. Membranes 12, 844. 10.3390/membranes12090844 36135863 PMC9505860

[B60] Tallmadge D. H. Huebner J. S. Borkman R. F. (1989). Acrylamide quenching of tryptophan photochemistry and photophysics. Photochem Photobiol. 49, 381–386. 10.1111/j.1751-1097.1989.tb09183.x 2727078

[B61] Thomas A. S. Elcock A. H. (2007). Molecular dynamics simulations of hydrophobic associations in aqueous salt solutions indicate a connection between water hydrogen bonding and the hofmeister effect. J. Am. Chem. Soc. 129, 14887–14898. 10.1021/ja073097z 17994735

[B62] Tsai S.-T. Fields E. Xu Y. Kuo E.-J. Tiwary P. (2022). Path sampling of recurrent neural networks by incorporating known physics. Nat. Commun. 13, 7231. 10.1038/s41467-022-34780-x 36433982 PMC9700810

[B63] Umetsu M. Tsumoto K. Nitta S. Adschiri T. Ejima D. Arakawa T. (2005). Nondenaturing solubilization of β2 microglobulin from inclusion bodies by l-arginine. Biochem. Biophysical Res. Commun. 328, 189–197. 10.1016/j.bbrc.2004.12.156 15670769

[B64] Upadhyay V. Singh A. Jha D. Singh A. Panda A. K. (2016). Recovery of bioactive protein from bacterial inclusion bodies using trifluoroethanol as solubilization agent. Microb. Cell Fact. 15, 100. 10.1186/s12934-016-0504-9 27277580 PMC4898390

[B65] Usmani Z. Sharma M. Tripathi M. Lukk T. Karpichev Y. Gathergood N. (2023). Biobased natural deep eutectic system as versatile solvents: Structure, interaction and advanced applications. Sci. Total Environ. 881, 163002. 10.1016/j.scitotenv.2023.163002 37003333

[B66] Villaverde A. Corchero J. L. Seras-Franzoso J. Garcia-Fruitós E. (2015). Functional protein aggregates: Just the tip of the iceberg. Nanomedicine 10, 2881–2891. 10.2217/nnm.15.125 26370294

[B67] Wakayama R. Uchiyama S. Hall D. (2019). Ionic liquids and protein folding—Old tricks for new solvents. Biophys. Rev. 11, 209–225. 10.1007/s12551-019-00509-2 30888574 PMC6441443

[B68] Walther C. Mayer S. Sekot G. Antos D. Hahn R. Jungbauer A. (2013). Mechanism and model for solubilization of inclusion bodies. Chem. Eng. Sci. 101, 631–641. 10.1016/j.ces.2013.07.026

[B69] Walther C. Mayer S. Trefilov A. Sekot G. Hahn R. Jungbauer A. (2014). Prediction of inclusion body solubilization from shaken to stirred reactors: Prediction of Inclusion Body Solubilization. Biotechnol. Bioeng. 111, 84–94. 10.1002/bit.24998 23860724

[B70] Walther C. Voigtmann M. Bruna E. Abusnina A. Tscheließnig A. Allmer M. (2022). Smart process development: Application of machine-learning and integrated process modeling for inclusion body purification processes. Biotechnol. Prog. 38, e3249. 10.1002/btpr.3249 35247040

[B71] Yadav N. Venkatesu P. (2022). Current understanding and insights towards protein stabilization and activation in deep eutectic solvents as sustainable solvent media. Phys. Chem. Chem. Phys. 24, 13474–13509. 10.1039/D2CP00084A 35640592

[B72] Yu Z. Reid J. C. Yang Y.-P. (2013). Utilizing dynamic light scattering as a process analytical technology for protein folding and aggregation monitoring in vaccine manufacturing. J. Pharm. Sci. 102, 4284–4290. 10.1002/jps.23746 24122727

[B73] Zimmerman M. I. Porter J. R. Ward M. D. Singh S. Vithani N. Meller A. (2021). SARS-CoV-2 simulations go exascale to predict dramatic spike opening and cryptic pockets across the proteome. Nat. Chem. 13, 651–659. 10.1038/s41557-021-00707-0 34031561 PMC8249329

