# Corrigendum: State-of-the-art and novel approaches to mild solubilization of inclusion bodies

**DOI:** 10.3389/fbioe.2024.1392514

**Published:** 2024-03-12

**Authors:** Robert Klausser, Julian Kopp, Eva Prada Brichtova, Florian Gisperg, Mohamed Elshazly, Oliver Spadiut

**Affiliations:** ^1^ Research Division Integrated Bioprocess Development, Institute of Chemical, Environmental and Bioscience, Vienna, Austria; ^2^ Christian Doppler Laboratory IB Processing 4.0, Technische Universität Wien, Vienna, Austria

**Keywords:** inclusion bodies, mild solubilization, ionic liquids, molecular dynamics simulation, refolding, aggregates

In the published article, there was an error regarding the **Affiliations** for Robert Klausser, Julian Kopp, Eva Prada Brichtova, Florian Gisperg, Mohamed Elshazly, Oliver Spadiut.

As well as having **Afiliation 1**, they should also have “Christian Doppler Laboratory IB Processing 4.0, Technische Universität Wien, Vienna, Austria”.

The authors apologize for this error and state that this does not change the scientific conclusions of the article in any way. The original article has been updated.

